# Notes and News

**Published:** 1905-03-11

**Authors:** 


### Notes and News.

The Duchess of Albany, as president of the Ladies' Association, attended a reception at the Royal Waterloo Hospital for Children and Women on February 28th.

It is proposed to add a children's ward to the Hospital of St. Cross, at Rugby.

A new dental and a new eye department are proposed at St. John's Hospital, Twickenham.

Belfast has now for the first time a Medical High Sheriff in the person of Dr. H. O'Neill. He has shown his appreciation of the honour by contributions to numerous local charities.

We pointed out in our leading article of February 11th that the leaders of the present American Mission relied for success largely on an appeal to undisciplined emotions rather than to sober judgment and knowledge. This opinion is justified by a letter from Dr. Torrey, which has appeared in the press. In this letter he writes authoritatively of vivisection, "I think it is a cruel practice, that absolutely no good has ever come of it, and that it brutalises many who practise it." We do not know whether this statement is more remarkable for its want of knowledge or its lack of charity.

The report of the Sydney Board of Health on a third outbreak of plague in that city gives an object lesson on the economic and other advantages of careful and continued observations. For nearly a year a rat staff was continuously at work exterminating thousands of rats, all of which were examined in the laboratory. At the end of 10 months one rat was found to be affected by bubonic plague, and prompt and stringent measures were taken to prevent infection being conveyed from the quarters where the rat was found. By thus adopting precautionary measures at the earliest possible opportunity, the authorities were enabled to prevent what might otherwise have proved an extensive and disastrous epidemic.

At the annual meeting of the Manchester Children's Hospital, at Pendlebury, a new out-patients' department was proposed.

As an outcome of the prosecution of a Leeds druggist for the sale of patent medicine containing poison to persons unknown, the sale of Easton's Syrup has been raised, but no decision has yet been arrived at in this connection.

Extensive alterations and improvements have been effected in the out-patients' department at the Chester Infirmary. The cost has been defrayed from the estate of the late J. A. Hay, Esq., of Cheltenham, who left money at discretion to his trustees.

At the Quarterly Court of the London Hospital held last week, it was stated that it is hoped that the scaffolding will be removed, and the scheme of structural improvements practically completed, by July in the present year. The entire scheme consists of a new nursing home, containing 275 separate bedrooms; the new laundry; the new out-patients' and pathological departments; new isolation block, with 85 beds, for infectious cases; new maternity and Hebrew wards; new operating theatres, of which there are now eight instead of two; new kitchens; and, in addition, the wings east and west have been gutted on the inside, ceilings and floors raised—necessitating a great deal of work in underpinning—and a new storey added to each wing.

We quote the following from the *Times*:—" At a meeting of managers of the Edinburgh Royal Infirmary on Monday, Colonel Warburton, the superintendent, stated that a deputation from the London Hospital visited the Royal Infirmary on January 19th to inquire into arrangements, and to discover, if possible, the secret of the economy practised there. On the 1st instant there was a meeting of the governors of the London Hospital, presided over by Mr. Sydney Holland, who reported the result of the deputation which had visited certain northern hospitals. He indicated a few items from one of the hospitals visited, which he named, which had a great advantage over the London hospitals. Meat in this hospital cost 3¾d. per lb., whereas in London it cost 4¾d or 5d. They gave no medicines to their patients, window cleaning cost £20 a year, against £300 in London, and no pensions were paid. Colonel Warburton replied to these statements, and showed that meat in 1904 cost the Royal Infirmary over 6¼d. per lb. They paid 10½d. per gallon for their milk, as compared with 8¾d. paid by the London Hospital. They gave their patients tea, coffee, sugar, and butter. The London Hospital did not. The cost of provisions per bed in the London Hospital was £31 5s. for 1903. In the Royal Infirmary for the same year it was £20 11s. 8½d., and in 1904 it was £19 16s. 3d. They paid £273 on account of pensions in 1904, and the wages of the window-cleaning staff amounted to £226. They gave medicines to all their in-patients, and supplied dressings to all their surgical out-patients."

